# Kcs1 and Vip1: The Key Enzymes behind Inositol Pyrophosphate Signaling in *Saccharomyces cerevisiae*

**DOI:** 10.3390/biom14020152

**Published:** 2024-01-26

**Authors:** Larisa Ioana Gogianu, Lavinia Liliana Ruta, Ileana Cornelia Farcasanu

**Affiliations:** 1 Doctoral School of Biology, Faculty of Biology, University of Bucharest, Splaiul Independenței 91-95, 050095 Bucharest, Romania; gogianu.larisa-ioana@s.bio.unibuc.ro; 2 National Institute for Research and Development in Microtechnologies, Erou Iancu Nicolae Str. 126A, 077190 Voluntari, Romania; 3 Faculty of Chemistry, University of Bucharest, Panduri Road 90-92, 050663 Bucharest, Romania; lavinia.ruta@chimie.unibuc.ro

**Keywords:** inositol pyrophosphate, inositol hexakisphosphate kinase, *Saccharomyces cerevisiae*, Kcs1, Vip1

## Abstract

The inositol pyrophosphate pathway, a complex cell signaling network, plays a pivotal role in orchestrating vital cellular processes in the budding yeast, where it regulates cell cycle progression, growth, endocytosis, exocytosis, apoptosis, telomere elongation, ribosome biogenesis, and stress responses. This pathway has gained significant attention in pharmacology and medicine due to its role in generating inositol pyrophosphates, which serve as crucial signaling molecules not only in yeast, but also in higher eukaryotes. As targets for therapeutic development, genetic modifications within this pathway hold promise for disease treatment strategies, offering practical applications in biotechnology. The model organism *Saccharomyces cerevisiae*, renowned for its genetic tractability, has been instrumental in various studies related to the inositol pyrophosphate pathway. This review is focused on the Kcs1 and Vip1, the two enzymes involved in the biosynthesis of inositol pyrophosphate in *S. cerevisiae*, highlighting their roles in various cell processes, and providing an up-to-date overview of their relationship with phosphate homeostasis. Moreover, the review underscores the potential applications of these findings in the realms of medicine and biotechnology, highlighting the profound implications of comprehending this intricate signaling network.

## 1. Introduction

In the intricate landscape of cellular signaling, the inositol pyrophosphate pathway was found to orchestrate a plethora of essential cellular processes, including cell cycle progression [1], cell growth [2], endocytosis and exocytosis [3,4], apoptosis, telomere elongation [4,5,6], ribosome biogenesis [7,8], and stress response [9], with more complex roles still being discovered, especially among higher eukaryotes [10]. Recently, inositol pyrophosphates became of interest in pharmacology and medicine due to their potential role as signaling molecules, and their involvement in various cellular processes ignited the interest in exploiting them as targets for therapeutic development [11,12]. Genetic modifications of the inositol pyrophosphate pathway hold promise for innovative disease treatment strategies, while concurrently offering practical applications in biotechnology [13]. The budding yeast *Saccharomyces cerevisiae*, a model organism revered for its genetic tractability and evolutionary conservation, has provided invaluable insights into the fundamental workings of this pathway, in which the enzymes Kcs1 and Vip1 play a central role.

**Figure 1:**
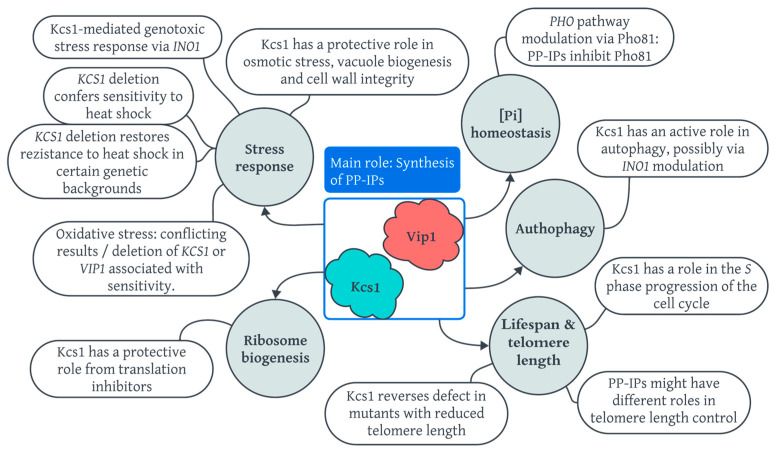
The multiple roles of Kcs1 and Vip1 in the cell processes of *Saccharomyces cerevisiae*.

This review seeks to bring together the existing knowledge on the role of the enzymes Kcs1 and Vip1, the inositol pyrophosphate synthases in *Saccharomyces cerevisiae*, and, implicitly, the signaling roles of their metabolites in the inositol pyrophosphate pathway (Figure 1). It includes their molecular characterization and their involvement in various cell processes, with an up-to-date landscape of the relationship with phosphate homeostasis. Furthermore, the review argues for the potential applications of these findings in the fields of medicine and biotechnology, illustrating the far-reaching impact of understanding this intricate signaling network.

A review on the roles of Kcs1 and Vip1 in cellular life is implicitly a discussion of the signaling roles of different inositol pyrophosphate species, which are the products of Kcs1 and Vip1. Since the notation of these metabolites varies in the reference literature, a quick clarification on notation is called for. As such, “PP-IP” refers generally to inositol pyrophosphate species, while “IP_6_”, “IP_7_” and “IP_8_” refer to inositol hexakisphosphate, inositol heptakisphosphate (diphosphoinositol pentakisphosphate) and bis-diphosphoinositol tetrakisphosphate, respectively. When distinguishing between IP_7_ species, the position of the pyrophosphate is indicated (e.g., “1PP-IP_5_”, “5PP-IP_5_”); likewise, for IP_8_ “1,5PP-IP_4_” is used. As Kcs1 and Vip1 mediate the synthesis of IP_7_ from IP_6_, they are sometimes referred to as yeast IP_6_ kinases (IP6K) or, more generally, as inositol pyrophosphate synthases.

## 2. *Saccharomyces cerevisiae* Kcs1 and Vip1 Overview: Structure; Localization; Regulation

### 2.1. Molecular Structure and Domains

**Figure 2:**
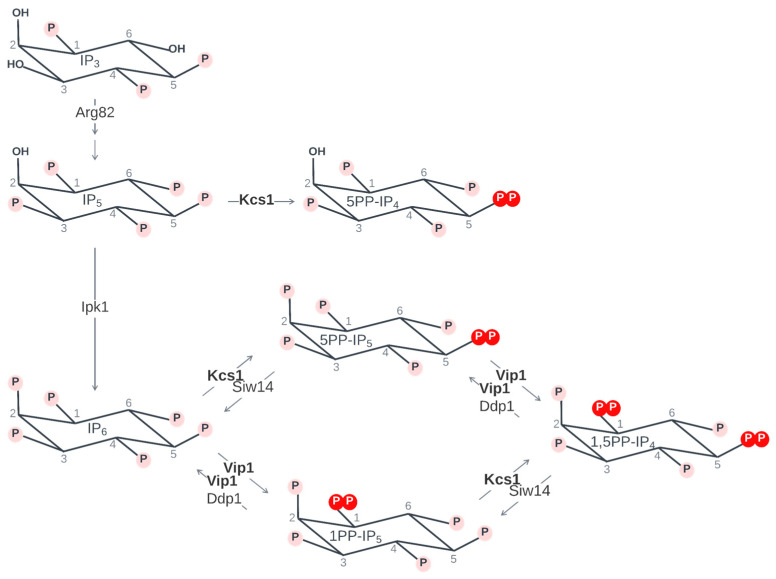
Synthesis of PP-Ips in yeast: In *Saccharomyces cerevisiae*, Kcs1 and Vip1 are the only inositol hexakisphosphate kinases (IP6K) described to date, both synthesizing PP-Ips primarily from IP_6_. Kcs1 adds a phosphate group at position 5, participating in the synthesis of 5PP-IP_4_ from IP_5_, 5PP-IP_5_ from IP_6_, and 1,5PP-IP_4_ from 1PP-IP_5_. Kcs1 action is reversed by Siw16 pyrophosphatase. Vip1 acts both as a kinase and pyrophosphatase, adding and removing phosphate from position 1; as a kinase, Vip1 participates in the synthesis of 1PP-IP_5_ from IP_6_ and 1,5PP-IP_4_ from 5PP-IP_5_. The kinase activity of Vip1 is reversed by Ddp1 pyrophosphatase (based on [1,20]).

The *KCS1* gene was discovered in 1995 [14], as part of a study that aimed to identify regulatory components of the Pkc1p-dependent pathways for growth and recombination control in *Saccharomyces cerevisiae* [13]. Through a genetic screening of mutants suppressing recombination, *KCS1* was found to code for a protein of 1050 amino acids, with a sequence resembling the leucine zipper family of transcription factors. It was hypothesized that Kcs1 has pleiotropic activity, and the multifunctional nature of Kcs1 was emphasized by identifying the different domains of the protein [15]. The first analyses on the kinase activity of Kcs1 through cloning techniques showed that the protein was able to stoichiometrically convert [^3^H]IP_6_ to IP_7_, thus confirming the IP_6_ kinase activity of Kcs1 [16]. Kcs1 adds a phosphate to the 5th position of the inositol polyphosphate ring, thus participating in the synthesis of various inositol pyrophosphates, such as 5-diphosphoinositol pentakisphosphate (5PP-IP_5_), 1,5-bis-diphosphoinositol tetrakisphosphate (1,5PP-IP_4_), and 5-diphosphoinositol tetrakisphosphate (5PP-IP_4_) [15,17] (Figure 2). The kinase activity of Kcs1 is reversed by Siw14, an inositol pyrophosphatase that hydrolyzes the β-phosphate from 5-diphospho-position [18] (Figure 2).

The existence of a second IP6K or inositol pyrophosphate synthase Vip1 (named Ids1 at the time) was suggested by York et al. in 2005 [5], when IP_7_ molecules were identified in *kcs1*Δ*ddp1*Δ double-knockout mutants. In a study conducted by Mulugu et al. in 2007, an analysis of the Vip1 protein sequence identified two conserved domains: an ATP-grasp domain located at the amino-terminal, exhibiting IP_6_ kinase activity, and a histidine acid-phosphatase domain situated at the C-terminus. To explore Vip1’s role in the synthesis of PP-IPs, yeast strains lacking the *KCS1*, *DDP1*, and *VIP1* genes were generated, which led to a depletion of IP_7_. However, upon expression of the *VIP1* gene in the defective mutants, the production of IP_7_ was restored, providing clear evidence that Vip1 was responsible for generating IP_7_ in vivo [19]. The kinase activity of Vip1 is reversed by Ddp1, an inositol pyrophosphatase that hydrolyzes the β-phosphate from the 1-diphospho-position [18] (Figure 2).

A more recent study [20] confirmed that Vip1 is a dual-function protein, working as a kinase but also displaying pyrophosphatase activity. As such, it was confirmed that Vip1 possesses two distinct domains with opposite roles: a kinase domain involved in the synthesis of 1PP-IP_5_ and 1,5PP-IP_4_ by preferentially adding a phosphate group to the 1 position of the inositol ring, and a pyrophosphatase that also exhibits selectivity for the β-phosphate found in 1 (Figure 2). This implies a switch in the role of the Vip1 protein in controlling the PP-IPs level.

### 2.2. Subcellular Localisation

The cytosolic localization of the Kcs1 and Vip1 in the yeast cell was experimentally determined through a high-throughput direct assay (HDA) [21]. This experimental evidence was further supported by computation analyses employing the inference from biological aspect of ancestor (IBA) method, which utilizes phylogenetic analyses and leverages the concept of common ancestry to infer the localization and activity of gene products [22]. According to the *Saccharomyces Genome Database* (SGD), IBA analyses suggested activity and localization in the cytoplasm for both Kcs1 and Vip1, as well as in the cell nucleus for Kcs1 [23]; for the nucleus localization, further experimental evidence is required. Vip1 was also found in the nucleus, associated with the nucleosome assembly factor Asf1 [24] and Spt5 [25], as demonstrated through immunoprecipitation assays and mass spectrometry analyses.

### 2.3. Transcription Regulation

Quantitative reverse transcription polymerase chain reaction (qRT-PCR) analysis provided evidence that the Gcr1 transcription factor functions as a positive regulator of the transcriptional activation of the *KCS1* gene in *S. cerevisiae* [26]. This regulatory mechanism comes into play during the cellular response to inositol starvation, thus pointing to a potential role of Gcr1 in orchestrating the transcriptional response of *S. cerevisiae* under conditions of inositol deprivation, also involving the inositol pyrophosphate signaling pathway.

The transcriptional control of the *VIP1* gene during the cellular response to stress in *S. cerevisiae* was highlighted in several experimental setups. Firstly, DNA-to-cDNA expression microarray experiments demonstrated that Sfp1 functions as a transcription factor for *VIP1* expression under stress conditions [27]. Further, the role of Stp1 and Xbp1 transcription factors regulating *VIP1* gene expression during cellular response to heat stress was elucidated through chromatin immunoprecipitation (ChIP) assay [28]. Collectively, these studies provide preliminary insights into the intricate regulatory network governing the transcription of *VIP1* in response to diverse stress conditions in *S. cerevisiae*. Additionally, mass spectrometry analysis indicated the involvement of protein modifiers such as Cdc28 and the serine/threonine protein phosphatase PP2A variant 1 in regulating the activity of the Vip1 protein [29,30].

## 3. Role of Kcs1 and Vip1 in Cellular Processes

### 3.1. Modulation of Phosphate Homeostasis

The pyrophosphate synthase function of Kcs1 and Vip1 highlights their significance in generating the key molecular messengers known as inositol pyrophosphates (PP-IPs). These molecules are now being studied for their significant participation in cellular signaling cascades.

The regulation of phosphate homeostasis is a vital cellular process that ensures the optimal utilization of an essential nutrient while preventing the toxicity caused by its excess. One player in this regulatory network is the PP-IP signaling pathway, which was found to participate in the activation and repression of the phosphate-responsive signaling pathway (*PHO* pathway) [31,32,33]. However, discrepancies across studies regarding the precise role of PP-IPs in *PHO* pathway modulation have prompted a re-evaluation of the underlying mechanisms. One notable source of discrepancy concerns the methods of analysis employed to quantify PP-IP levels. Traditional high-performance liquid chromatography (HPLC)-based approaches have frequently been used, but their inability to discriminate between some PP-IP species, particularly to distinguish between IP_7_ and IP_8_ species, has potentially led to inconsistent findings regarding the identification of the PP-IP species involved in *PHO* regulation and their mechanisms of action.

The link between *KCS1* and phosphate regulation was first suggested in 1995 [14] and further explored in 2005 [31], when a screening of a collection of yeast deletion strains suggested that Kcs1, among Arg82, Plc1, and Adk1, is involved in the negative regulation of the *PHO* pathway upstream of Pho81. Moreover, while Plc1, Arg82, and Kcs1 were all known to be involved in the synthesis of polyphosphates, only the overexpression of *KCS1* revealed a suppressing effect of *PHO5* expression under low-phosphate conditions. Conversely, in a more recent study, the lack of *KCS1* gene resulted in high levels of Pho81, even under high-phosphate conditions [34].

*VIP1* was also linked to the regulation of the *PHO* pathway when Lee et al. [32] proposed that the form of IP_7_ generated by Vip1, namely 1PP-IP_5_, which is distinct from the 5PP-IP_5_ produced by Kcs1, plays a crucial role as a regulator of the Pho80–Pho85 cyclin/cyclin-dependent kinase (CDK) complex, which is a key component of the *PHO* phosphate response pathway in yeast. In their study, Lee et al. employed a biochemical approach to identify the cellular components involved in controlling the response to phosphate starvation in cells grown under low-phosphate conditions. They found that the Pho80–Pho85 cyclin–CDK complex is active and phosphorylates transcription factor Pho4, causing it to be exported from the nucleus to the cytoplasm when phosphate levels are high. Conversely, during phosphate starvation, the Pho80–Pho85 complex is inhibited, leading to the accumulation of dephosphorylated Pho4 in the nucleus and the transcription of *PHO* genes. The Pho81 CDK inhibitor is bound to the Pho80–Pho85 complex and is required for its inhibition in response to phosphate limitation. Lee et al. identified IP_7_ as a molecule capable of inhibiting the kinase activity of the Pho80–Pho85 complex in a Pho81-dependent manner; moreover, the inactivation of Pho80–Pho85 by IP_7_ was thought to be specific, as other forms of inositol and its phosphorylated derivatives did not affect the kinase activity. To demonstrate the involvement of Vip1 in controlling the *PHO* pathway, Lee et al. tested extracts from phosphate-starved cells lacking Kcs1 and Vip1 kinases. Only extracts from cells lacking Kcs1 but containing Vip1 were able to inhibit the Pho80–Pho85 complex in a Pho81-dependent manner [32,35]. The role of Vip1 in the modulation of the *PHO* pathway has also been confirmed for another yeast species, *Schyzosaccharomyces pombe*, where the ortholog of *S. cerevisiae*’s VIP1, Asp1, has been shown to regulate the expression of *PHO* genes in a similar manner [36].

Consistent with the above findings, it was shown that Pho4 is responsible for initiating the transcription of antisense and intragenic RNAs within the *KCS1* locus [37]. These RNAs work to suppress the activity of Kcs1, where suppression is achieved by generating a shortened Kcs1 protein through a hybrid formation with the *KCS1* mRNA, along with the translation of the intragenic RNA. Consequently, IP_6_ becomes more accessible for Vip1. This allows for Vip1 to enhance its use of IP_6_ to produce 1PP-IP_5_, which is considered an essential IP_7_ for low-phosphate signaling [32]. The reduction in Kcs1 activity is significant because Kcs1 is capable of phosphorylating IP_7_ to produce IP_8_. By diminishing Kcs1’s activity, the accumulation of IP_7_ species is ensured, subsequently intensifying the effects of low-P_i_ signaling. According to Nishizawa et al., this sequence of events establishes a positive feedback loop that amplifies the low-P_i_ signaling process [37].

**Figure 3:**
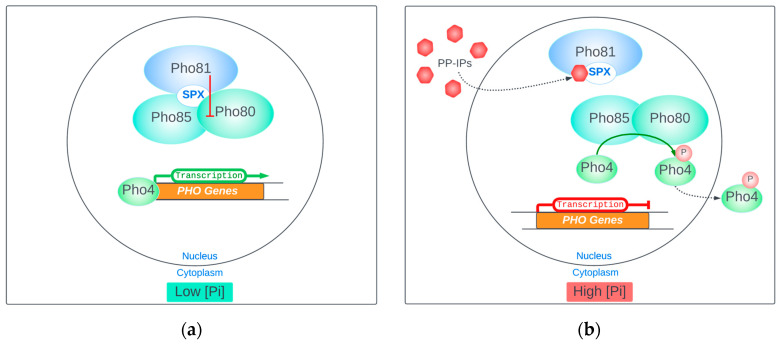
The *PHO* pathway modulation by PP-IPs: (**a**) in low-[P_i_] conditions, Pho81 inhibits the Pho85–Pho80 complex, leaving Pho4 free to activate the transcription of *PHO* genes; (**b**) in high-[P_i_] conditions, PP-IPs bind to Pho81, inhibiting its interaction with the Pho85–Pho80 complex; phosphorylated Pho4 is exported outside the nucleus and the *PHO* genes’ transcription remains inactivated (based on [32,33]).

For the PP-IP signaling mechanism, it was proposed that a high concentration of PP-IPs in the nucleus under high-phosphate conditions determines the binding of the PP-IPs to Pho81 (Figure 3), thus preventing its suppressing effect on the Pho80–Pho85 cyclin–CDK complex [32]. The binding site of PP-IPs to Pho81 was thought to be the so-called “minimum domain”, which is a small amino-acid fragment, supposedly of Pho81, which is sufficient to regulate the Pho80–Pho85 complex [38,39].

The hypothesis that 1PP-IP_5_ is the main signaling PP-IP in the *PHO* pathway has been challenged recently [33], in a study that utilized capillary electrophoresis coupled with mass spectrometry (CE-MS) to analyze the PP-IP profile in both wild-type (WT) *S. cerevisiae* and mutants within the inositol pyrophosphate pathway. This approach enabled a finer-resolution analysis in the effort to highlight which of the PP-IPs is involved in the modulation of the *PHO* pathway. The results of this comprehensive investigation offered several intriguing insights. Firstly, the study corroborated an earlier finding [40] of a marked decline in all PP-IP species under conditions of phosphate starvation; by monitoring the levels of the different PP-IPs, it found a near-complete depletion observed in 1PP-IP_5_, 5PP-IP_5_, and 1,5PP-IP_4_. This observation underscores the potential importance of PP-IPs in responding to changes in P_i_ availability. More significantly, the study revealed a strong correlation between the decline in PP-IP levels and the P_i_ starvation response through the *PHO* pathway. This correlation was not only evident during P_i_ starvation but also following the deletion of IP6Ks, suggesting that the PP-IP–*PHO* pathway connection is intrinsic and independent of specific genetic backgrounds. Further analysis into the levels of each of the PP-IP species demonstrated that neither of the IP_7_ species (1PP-IP_5_ or 5PP-IP_5_) are critical factors in the *PHO* pathway activation. Instead, 1,5-IP_4_ emerged as the PP-IP species were most consistently correlated with *PHO* pathway activation across different mutants and conditions. Considering these findings, the study’s proposed model offers a novel perspective on the mechanism of *PHO* pathway modulation by PP-IPs. The study suggests that P_i_ starvation is signaled by a decrease in PP-IPs levels rather than an increase and, contrary to earlier assumptions [32,37], the key signaling molecule identified is 1,5PP-IP_4_, rather than the previously suggested 1PP-IP_5_. Moreover, the study highlights the significance of the *N*-terminal *SPX* functional domain of *PHO81*, implicating this as the primary receptor responsible for PP-IP-mediated control over the *PHO* pathway. This revelation shifts attention from the central minimal domain of PHO81 [38,39], introducing a new focal point in understanding the PP-IPs–*PHO* pathway interaction.

The revised working model proposed by Chabert et al. [33] offers a comprehensive framework for *PHO* pathway modulation. Under conditions of sufficient cytosolic P_i_, the accumulation of 1,5PP-IP_4_ binding to the SPX domain of Pho81 is proposed to inhibit the interaction between Pho81 and Pho85–Pho80, resulting in the activation of the Pho85–Pho80-mediated phosphorylation of Pho4. This phosphorylated Pho4 relocates to the cytosol, leading to the repression of the *PHO* pathway. Conversely, under P_i_ starvation, the decline in 1,5PP-IP_4_ levels releases the *SPX* domain of Pho81. Active Pho81, in turn, inhibits Pho85-Pho80, allowing for Pho4 to accumulate in the nucleus and activate the *PHO* pathway. The study’s comprehensive analysis has provided critical insights into the nuanced role of PP-IPs in *PHO* pathway modulation. However, it is premature to conclude that all the discrepancies with earlier studies have been resolved. Further research in this direction is necessary to offer a refined and consensual model for the interaction between PP-IPs and the *PHO* pathway, which is essential for a deeper understanding of cellular phosphate homeostasis and signaling.

### 3.2. Cellular Stress Response

The budding yeast’s response to a stressful environment necessitates comprehensive shifts in global gene expression, alongside the precise regulation of protein translation and enzymatic activities. Research into genomic expression patterns in *S. cerevisiae* has unveiled a remarkable assembly of approximately 900 genes engaged in the environmental stress response (ESR), which encompasses around 300 genes that are induced and approximately 600 genes that are repressed, all orchestrated when cells are confronted with challenging growth conditions like heat shock, exposure to toxins, osmotic fluctuations, or nutrient deprivation [41]. Recent investigations propose a role for PP-IPs in pivotal biological processes that underpin cellular adaptability to environmental stressors [15,42]. Although the precise mechanisms through which they achieve this remain only partially elucidated, several hypotheses have been put forth [2,9]. While the involvement of PP-IPs in cell stress response has been reviewed in detail by Morrissette et al. [9], this section discusses the main ideas and up-to-date findings concerning the relationship between *KCS1*, *VIP1*, and the response to heat shock, oxidative stress, genotoxicity, osmotic stress, vacuolar formation, and cell integrity.

#### 3.2.1. Heat Shock

One of the first studies on *KCS1* suggested a possible protective role in cellular stress response, since *kcs1*Δ mutants suppressed a growth defect that manifested at 34 °C by strains with a protein kinase C allelic variant (*pkc1-4*) [14]. The absence of some PP-IPs is probably not protective under high-temperature conditions per se, but only corrective of certain genetic backgrounds, such as the presence of different *PKC1* allele variants, whose activity is possibly modulated by PP-IPs. This conclusion is backed up by subsequent studies on the involvement of PP-IPs in heat response. The deletion of *KCS1* was found to confer high sensitivity to heat in a screening of non-essential deletion mutants [43]; the same screening showed that the deletion of the inositol pyrophosphatase *SIW14* had a protective role under high-temperature conditions. Although the screening was not followed by the profiling of PP-IPs, since the deletion of the kinase *KCS1* and phosphatase *SIW14* resulted in the opposite effects regarding resistance to high temperatures, it was suggested that high levels of PP-IPs have a protective role in heat shock. Another study confirmed this conclusion, showing that the *S. cerevisiae siw14*Δ mutants were significantly more resistant to heat shock than wild-type cells, having survived incubation at 50 °C. Moreover, when the *SIW14* gene was restored in the mutant strains, they recovered their sensitivity to high temperatures and presented a similar phenotype to the wild type [42].

#### 3.2.2. Oxidative Stress

The PP-IPs pathway is also involved in resistance to oxidative stress, although the role of PP-IPs in the adaptation to oxidative stress is unclear due to the conflicting experimental data in the recent literature. Reactive oxygen species (ROS) molecules, such as hydrogen peroxide (H_2_O_2_), are important signaling molecules at low concentrations, but are also highly toxic at high concentrations [44]. In yeast, H_2_O_2_ exposure can lead to DNA-base modifications or breaks in the DNA molecule, which can trigger DNA-checkpoint responses to activate DNA repair mechanisms; at very high concentrations, exposure to H_2_O_2_ leads to cell death [45]. When exposed to various concentrations of H_2_O_2_ ranging from 0.1 mM to 1 mM, *S. cerevisiae* showed a significant increase in the survival rate (evaluated by colony formation assays) in *kcs1*Δ mutants compared to the wild type [46]; since *vip1*Δ mutants also displayed resistance to H_2_O_2_, it was hypothesized that 1,5PP-IP_4_ might be the main mediating molecule in the response to H_2_O_2_-induced stress. On the other hand, Steidle et al. [42] showed that *siw14*Δ mutants were more resistant to H_2_O_2_ treatment than the wild-type strain (40% vs. 2%). When the PP-IP levels were measured, the wild-type strain presented increased levels of IP_7_ and IP_8_ under stress conditions, while the *siw14*Δ mutants presented an expected higher level of PP-IPs than the wild type, but no significant increase in PP-IP levels was found under stressful growth conditions. Moreover, the same study showed, through microarray and RT-qPCR assays, that the ESR is partially induced in unstressed *siw14*Δ mutants, suggesting that high levels of PP-IPs activate the ESR and induce resistance to oxidative stress. A global gene expression study also confirmed that *kcs1*Δ and *vip1*Δ mutants are sensitive to oxidative and osmotic stresses [2].

#### 3.2.3. Genotoxic Stress

The involvement of PP-IPs in cellular response to genotoxic stress is underscored by a series of interconnected findings. The focal point of this argument is Opi1, studied for its potential to mediate genotoxic stress. A recent study [47] revealed that in *opi1*Δ, the expression of *INO1*, involved in the synthesis of inositol phospholipids and inositol polyphosphates, exhibited a significant increase upon exposure to the genotoxic agent methyl methane sulfonate (MMS). This induction of *INO1* expression is attributed to the activation mediated by Ino2-Ino4 [48], transcriptional activators whose levels surge in the absence of Opi1. Notably, *INO1*’s regulatory framework was shown to pivot on Kcs1, implying Kcs1-dependent modulation. This leads to the hypothesis that PP-IPs synthesized by Kcs1 might have a functional role in recruiting Ino2-Ino4 to the promoter region of *INO1*. The study’s line of reasoning was validated by a genetic rescue experiment, wherein the deletion of *KCS1* in an *opi1*Δ knockout strain successfully mitigated the MMS sensitivity that typically manifests in the absence of Opi1. This outcome implies a contrasting function for Opi1 and PP-IPs in the modulation of Ino2-Ino4 action [47].

#### 3.2.4. Osmotic Stress, Vacuolar Biogenesis, Cell Integrity

The specific effects of the complete deletion of the *KCS1* gene and the deletion of specific domains within the gene on cellular processes, particularly vacuolar biogenesis and cell wall structure, were investigated [15]. Firstly, the deletion of the *KCS1* gene led to a drastic decrease of 93% in the levels of 5PP-IP_5_ and 1,5-IP_4_ compared to the wild-type cells, confirming that the *KCS1* gene plays a crucial role in the synthesis of these inositol pyrophosphates; a similar observation had been previously reported by Saiardi et al., with a decrease of about 80% in diphosphoinositol pyrophosphate levels after the disruption of *KCS1*’s kinase domain, compared to the wild type [17]. The mutant phenotype exhibited notable alterations in cellular structures. Specifically, the vacuolar compartment, which is responsible for storage and degradation within the cell, appeared small and fragmented in comparison to the single large vacuole typically observed in wild-type cells [15,17]. This fragmentation suggests that the absence of the *KCS1* gene affects vacuolar morphogenesis. Furthermore, the mutant strains had a normal response to osmotic stress, indicating their ability to cope with changes in sorbitol concentrations; however, their resistance to salt stress was compromised, since at NaCl concentrations of 0.6 M or 0.8 M, the mutant strains showed an inhibition of colony formation, suggesting their heightened sensitivity to high salt concentrations [15]. Additionally, a leakage of intracellular alkaline phosphatase in the mutant strain was observed, indicating a defect in the maintenance of the cell wall integrity. The observed defect highlights the importance of the *KCS1* gene in cell wall stability. In further validation of the Kcs1’s role as an inositol pyrophosphate synthase, the *kcs1*Δ mutants were also transformed with a plasmid containing a catalytically inactive form of Kcs1. Once again, the resulting phenotype presented structural defects and sensitivity to high concentrations of salt. These results might raise the question as to whether the response to stress is indeed mediated by PP-IPs, or just a consequence of the structural defects of the mutant cells. Interestingly, when another domain of the Kcs1 protein was modified, specifically the leucine zipper motifs, the vacuolar morphogenesis was mildly affected, and the cell wall stability was still severely compromised without affecting the resistance to salt stress. This result supports the hypothesis that the response to stress is dependent upon the PP-IP levels and not merely a result of structural cell damage. Moreover, this result indicated that the leucine zipper motifs of Kcs1 also play a role in maintaining proper vacuolar structure and cell wall integrity and supports the idea that Kcs1 has pleiotropic activity. Overall, these findings demonstrate the significance of the *KCS1* gene in regulating inositol pyrophosphate levels, vacuolar morphogenesis, salt stress resistance, and cell wall integrity, and highlight the need for further exploration into the mechanisms underlying these pleiotropic effects.

### 3.3. Involvement in Fundamental Cell Processes

#### 3.3.1. Autophagy

Studies on autophagy reported that some PP-IP species might be involved in regulating this process [49]. To determine the role of the genes of the PP-IP pathway in autophagy, several knock-out mutants were transformed with the fusion protein GFP-Atg18 and monitored during nitrogen starvation [50]. Atg18 is a ubiquitin-like protein involved in membrane fusion and phagophore expansion during the formation of the autophagosome [51]; since ATG18 is more rapidly degraded by the vacuolar proteases than the GFP of the fused GFP-18 protein [50], the accumulation of GFP reflected autophagic reflux. The result of the study suggested that *arg28*Δ mutants had reduced autophagy, while *ipk1*Δ, *vip1*Δ, and *ddp1*Δ had similar levels of autophagy to the wild type. In contrast, *kcs1*Δ showed no measurable level of autophagy. Autophagy levels, quantified by analyzing the vacuolar alkaline phosphatase activity, were also reduced in *kcs1*Δ mutants, and microscopy studies suggested that Kcs1 is necessary for the correct localization of the pre-autophagosome structure (PAS) under nitrogen starvation [50].

In another study, which explored the functional role of Gcr1 under inositol starvation in autophagy, a link was found between the lack of expression of *GCR1* and the downregulation of *INO1*. However, further analysis showed that deleting *GCR1* reduced the expression of Kcs1 [26]. Since 5PP-IP_5_, synthesized by Kcs1, had been previously found to regulate the transcription of *INO1* [52], and *INO1* is involved in the correct formation of the autophagosome, it is possible that the defects in the *kcs1*Δ mutants may be due to the downregulation of *INO1* in the absence of 5PP-IP_5_.

#### 3.3.2. Lifespan and Telomere Length

*KCS1* was found to be the main kinase involved in the synthesis of pyrophosphates during the S phase of the cell cycle [1]; moreover, *kcs1*Δ mutants had a delayed cell cycle progression, whereas *ddp1*Δ mutants had an accelerated S phase progression, suggesting that PP-IPs might have an active role in the S phase of the cell cycle.

Phosphate is an important determinant for lifespan regulation in many organisms. In yeast, it was shown that high levels of intracellular IP_6_ reduce the lifespan [53]: research on knockout mutants of different secreted acid phosphatases (APases such as *PHO3*, *PHO5*, *PHO11*, *PHO12*) demonstrated a shorter lifespan, regardless of the levels of external phosphate compounds; to uncover the substrates through which APases exert their lifespan-regulatory effects, the study conducted a screening involving the disruption of enzymes in the polyphosphate pathway. The results revealed a connection between a shorter lifespan and higher IP_6_ levels. Specifically, disrupting *KCS1* significantly shortened the lifespan, while *VIP1* disruption amounted to no such effect, possibly due to its phosphatase activity. Further research on PP-IPs could provide more insight into their role in lifespan regulation.

Two independent studies investigating the regulation of telomere length and lifespan in *S. cerevisiae* proposed a connection between the PP-IPs pathway and telomere length regulation via Tel1 [5,6]. York et al. revealed a link between the overproduction of PP-IPs synthesized by Kcs1 and the shortening of telomeres in yeast, whereas the loss of these PP-IPs resulted in longer telomeres in the presence of Tel1. Saiardi et al. corroborated these findings and proposed that PP-IPs and Tel1 are part of the same signaling pathway of telomere regulation [6]. Another piece of research highlighted the involvement of *KCS1* in regulating telomere length while investigating the roles of very-long-chain fatty acid synthesis in the regulation of the telomere length and lifespan of *S. cerevisiae*. In this context, it was shown that the deletion of *ELO3*, a gene encoding for a fatty acid elongase, resulted in reduced telomere length; however, this defect was nearly completely reversed by the deletion of *KCS1* in *elo3*Δ mutants. It was hypothesized that the deletion of *KCS1* apparently restored the telomere binding and the telomere protective function of the Ku structure at the chromosome end, effectively fixing the telomere length issue [54]. Together, these results suggest that different PP-IPs might be involved in regulating elements that determine telomere length and lifespan.

#### 3.3.3. Ribosome Biogenesis

The *KCS1* gene, studied in relation with the Rrs1 protein, which plays an essential role in the assembly of the 60S ribosomal subunit in *S. cerevisiae*, suggested that the PP-IP metabolism affects ribosome biogenesis [7]. While the *rrs1-1* mutant expressed sensitivity to low temperatures, a *kcs1* allele variant resulting from the truncation of the kinase domain had a protective effect in *rrs1-1* mutants. Yeast cells lacking the Kcs1 kinase or expressing an inactive form exhibited a high sensitivity to translation inhibitors, which resulted in a decreased level of protein synthesis; it was found that these mutants displayed reduced rates of rRNA synthesis and, consequently, reduced the levels of ribosome subunits [8].

In summary, these findings establish a correlation between the PP-IP pathway and cellular processes on the one hand, and stress response on the other hand. However, ongoing research is also delving into less explored avenues related to PP-IPs. Gaining a comprehensive understanding of the PP-IP pathway, particularly the roles of IP6Ks and the signaling targets of distinct PP-IP species, holds significant potential for influencing how cells react to stressors and rectifying cellular abnormalities.

## 4. Perspectives for Applicative Fields

### 4.1. Therapeutic Strategies Involving PP-IPs

The inositol pyrophosphate pathway is highly conserved across eukaryotes, from yeast to plants and animals [19]. The yeast’s Kcs1 orthologs in mammals are known as inositol hexakisphosphate kinases (IP6Ks). In humans and mice, IP6Ks exist in three distinct isoforms [10] that share common functions, namely converting IP_6_ to 5PP-IP_5_ (IP_7_) and IP_5_ to 5PP-IP_4_, and also contribute to the synthesis of IP_8_ from 1PP-IP_5_. Similarly, the Vip1 orthologs in mammals are the PP-IP_5_ kinases (PPIP5Ks), with two isoforms identified in humans and mice (Table 1). Notably, yeast Vip1 and mammalian PPIP5Ks possess a dual functionality, featuring both a kinase and a phosphatase domain [55]. The kinase domain of PPIP5Ks converts IP_6_ to 1PP-IP_5_, and 5PP-IP_5_ to IP_8_. In humans an mice, IP_8_ is mostly synthesized from 1PP-IP_5_ by PPIP5Ks rather than from 1PP-IP_5_ by IP6Ks [56], which is probably why earlier models of the inositol pyrophosphate pathway [10] present the IP6Ks as the only inositol hexakisphosphate synthases and PPIP5Ks only as inositol diphosphoinositol pentakisphosphate synthases. Despite the conserved kinase domains, the expression profiles of IP6K and PPIP5K isoforms vary across tissues, adding an additional layer of complexity to the regulation of the inositol pyrophosphate pathway in different physiological contexts.

In recent years, the PP-IP pathway and the associations between PP-IPs with various cellular mechanisms have gained relevance for the medical field due to the supposed role of PP-IPs in the normal development of animals and their capacity to modulate and regulate biological functions. As such, PP-IPs constitute potential targets for novel therapeutic strategies. An analysis of PP-IP signaling on animal models revealed an association between defective PP-IP signaling and abnormal body development [56,58]. However, reducing PP-IPs by silencing IP6Ks was associated with a protective role in the occurrence of pathological conditions, represented mainly by metabolic diseases such as diabetes mellitus and obesity [59]. In addition, the modulation of the PP-IP pathway is of interest in oncology, due to their role in regulating cell cycle progress, proliferation, and programmed cell death [60]. For example, IP_5_ and IP_6_ have shown inhibitory properties towards cell proliferation and promote cancer cell apoptosis [61,62]. The knowledge transfer for advancing novel therapeutics through the PP-IP pathway entails two main strategies: the first involves modifying enzyme actions within the pathway to regulate PP-IP levels, thus influencing cellular signaling; the second strategy entails controlled exposure to specific PP-IP levels, directly modulating cellular processes. These strategies offer distinct approaches for leveraging the pathway’s potential in therapeutic development, and both are exemplified in the following.

Due to the pleiotropic activity of the IP6Ks and the complex network of interactions between PP-IPs and other signaling pathways, current research in the therapeutic roles of PP-IPs in oncology is obtaining some conflicting results. For instance, on the one hand, IP_7_ was identified as a significant mediator of cancer cell migration and promotor of metastasis by sequestration of the tumor-suppressor liver kinase B1 [63]. On the other hand, it was shown that IP_7_ regulates the activity of the p53 protein [64] and is required for p-53-mediated apoptosis in colorectal cancer cells [65]. Further research is required to resolve the role of IP6Ks and their metabolites in tumorigenesis and tumor suppression.

The study of the PP-IP pathway may provide valuable insights into alleviating neurodegenerative diseases [66,67,68]. One such disease is Alzheimer’s, a neurodegenerative condition characterized by the accumulation of amyloid plaques (composed of amyloid-beta peptides) and neurofibrillary tangles (composed of hyperphosphorylated Tau proteins) [69]. It was suggested that reducing the buildup of hyperphosphorylated Tau could slow down the progression of Alzheimer’s disease [34]: by investigating the connection between Tau phosphorylation and PP-IPs in humanized yeast models of Alzheimer’s, researchers discovered that cells lacking Kcs1 and Vip1 kinases exhibited higher levels of hyperphosphorylated Tau; additionally, disrupting the PP-IP pathway upstream of IP_6_ resulted in reduced levels of total Tau, but did not decrease the extent of hyperphosphorylation. It was suggested that defects in the inositol pathway may impact the activity of Pho85 in the *PHO* pathway, potentially explaining the hyperphosphorylation of Tau in *kcs1*Δ mutants. However, the same effect observed in *kcs1*Δ mutants suggests the presence of an additional mechanism that is independent of Pho85. Further studies are necessary to explore the relationship between PP-IPs and Alzheimer’s disease.

Modulating the activity of mammalian IP_6_ kinases, especially *IP6K1*, has been proposed as a potential therapeutic target for managing metabolic diseases [59], mostly based on preliminary results on mice models. It was found that the IP_7_ produced by *IP6K1* induces the secretion of insulin by β-cells and simultaneously inhibits the Akt protein kinase that signals insulin levels in metabolic tissues, leading to hyperinsulinemia and insulin resistance [70]. *IP6K1* also regulates fat accumulation via the AMPK-mediated adipocyte energy metabolism [71]. Conversely, the deletion of *IP6K1* offered protection to insulin resistance and reduced high-fat-diet obesity and fatty liver in mice. A successful disturbance of the PP-IP pathway by selective inhibitors for IP6Ks [72,73] was reported in mice, showing ameliorated symptoms of obesity, fatty liver, and insulin resistance [70].

It is important to note that mammalian cells, as opposed to yeast, have three different IP6Ks, each with their own expression profile, in different types of tissues, which can also vary from one organism to another [59,74]. This aspect should be considered when developing novel therapeutic strategies based on PP-IP modulation.

PP-IP-based therapeutics have also been discussed in relation to microbial pathogenicity. For instance, recent studies on PP-IPs in the fungus *Cryptococcus neoformans* reveal IP_7_’s importance in host adaptation, immunity, and pathogenicity. This highlights the polyphosphate biosynthesis pathway as a new virulence-related pathway [75,76,77]. Moreover, PP-IPs may participate in modulating filamentous growth in fungal pathogens such as *Candida albicans*: this hypothesis was proposed by a study on pseudo-hyphal growth in *S. cerevisiae*, where the profiling of PP-IP levels in homozygous diploid knockout mutants of *VIP1* and *KCS1* showed that IP_7_ isoforms are indicative of the pseudo-hyphal growth state [78]. This result holds significance for the study of fungal pathogens, as filamentous *S. cerevisiae* strains mimicking the directional growth of pathogenic fungi like *Candida albicans* serve as valuable models [79,80]. However, research on filamentous pathogenic fungi is necessary to confirm this hypothesis.

It is noteworthy that fungal kinases for PP-IP synthesis differ from human ones, making fungal-specific IP6K inhibitors a possible approach to antifungal drug development [75]. In contrast, developing human IP6K-specific inhibitors might be more challenging due to the high similarity between their functional domains [11]. Thus, modulating the PP-IP pathway in humans might be a more challenging approach in therapeutics.

### 4.2. Biotechnological Strategies Involving PP-IPs

PP-IPs are emerging as potential contributors to biotechnological advancements, as demonstrated by their influence on metabolic processes like *S*-adenosylmethionine (SAM) production and octanoic acid levels in yeast, offering promising avenues for exploration in this context.

The Gcr1 transcription factor controls the expression levels of several glycolytic genes in *S. cerevisiae*, and it has been proposed that its activity might regulated, in turn, by the levels of PP-IPs: when intracellular PP-IP levels rise, Gcr1 undergoes pyrophosphorylation; this modification weakens its interaction with Rap1 and Gcr2, leading to the reduced activation of glycolytic genes in the absence of the Rap1-Gcr1-Gcr2 transcription activator complex [81,82]. Successful attempts at the metabolic reprogramming of glycolysis through alterations in the inositol polyphosphate metabolism have been reported. For instance, the deletion of *ARG82*, *IPK1*, and *KCS1* in yeast resulted in an improved production of SAM; furthermore, the deletion of *KCS1* in a high-SAM-producing *S. cerevisiae* strain resulted in a 46.2% increase in SAM production [83].

A high-throughput screening of around 22,000 *S. cerevisiae* colonies was set up in the search for genes whose overexpression could improve octanoic acid levels in yeast [84], revealing that the co-overexpression of *FSH2* (a putative serine hydrolase) and *KCS1* in an octanoic acid producer strain led to increased octanoic acid titers and yield. The overexpression of *KCS1* by itself did not increase titers or yield, whereas the overexpression of only *FSH2* showed a 29% increase in titers. The role of *KCS1* in the synthesis of octanoic acid remains to be elucidated. However, while the study suggests that the octanoic acid titers are possibly enhanced due to the *KCS1* overexpression affecting glycolysis, conflicting results [83] suggested that the transcription levels of glycolytic genes are enhanced not by the overexpression of *KCS1*, but on the contrary, by *kcs1*Δ knockout. As such, the link between *KCS1* expression, pyrophosphate levels and glycolysis should be further investigated.

## 5. Conclusions

The Kcs1 and Vip1 have distinct positions on the signaling map of *S. cerevisiae*, and their action is indissolubly associated with the production of PP-IPs. While the involvement of Kcs1 and Vip1 in important processes is demonstrated, the exact sequence of events that occur from kinase activation to PP-IP action is still elusive.

The similarity in catalytic activities between yeast and mammalian IP_6_Ks implies an evolutionary conservation of their active site domains [85], making fundamental research on *S. cerevisiae* valuable for gathering further insights into the roles of IP6Ks and their products. While molecular cloning techniques have been crucial for uncovering the pleiotropic roles of the IP6Ks, the development of a cellular assay for the real-time, in vivo detection of PP-IPs would be a tremendous step in the understanding of PP-IP-mediated response. Moreover, establishing a streamlined method for synthesizing and recovering PP-IPs could provide ready access to these products for experimental procedures. Such advancements are essential for addressing critical questions: how does the modulation of PP-IP levels impact diverse cellular processes? Do PP-IPs function as signaling molecules only through allosteric interactions with various protein domains, through the nonenzymatic phosphorylation of proteins [81], or both? Further exploration of these avenues is needed to shed light on the functional implications of PP-IPs in cellular regulation and on their mechanisms of action.

## Figures and Tables

**Table 1:** Mammalian homologs of the yeast Kcs1 and Vip1.

*Homo sapiens*	Expression in Human Tissues	*Mus musculus*	Activity	*Saccharomyces cerevisiae*	Ref.
IP6K1	Ubiquitous.	Ip6k1	Inositol pyrophosphate kinase—adds a PP at C5.	Kcs1	[10,56,57]
IP6K2	Ubiquitous, higher levels in breasts, testis, colon, prostate, thymus, adipose tissue, smooth muscles.	Ip6k2			
IP6K3	The thyroid, heart muscle, skeletal muscles.	Ip6k3			
PPIP5K1	Ubiquitous, higher level in the heart muscle, skeletal muscles, the brain.	Ppip5k1	Inositol pyrophosphate kinase/phosphatase—adds/removes a PP at/from C1.	Vip1	[55,56,57]
PPIP5K2	Ubiquitous	Ppip5k2			
